# Clinical Effectiveness of Modified Laparoscopic Fimbrioplasty for the Treatment of Minimal Endometriosis and Unexplained Infertility

**DOI:** 10.1155/2015/730513

**Published:** 2015-05-06

**Authors:** Sarah E. Franjoine, Mohamed A. Bedaiwy, Faten F. AbdelHafez, Cuiyu Geng, James H. Liu

**Affiliations:** ^1^Division of Reproductive Endocrinology and Infertility, Department of Obstetrics and Gynecology, University Hospitals Case Medical Center, MacDonald Women's Hospital, Cleveland, OH 44106, USA; ^2^Division of Reproductive Endocrinology and Infertility, Department of Obstetrics and Gynaecology, The University of British Columbia, D415A, 4500 Oak Street, Vancouver, BC, Canada V6H 3N1; ^3^Women's Health University Center, Department of Obstetrics and Gynecology, Faculty of Medicine, Assiut University, Assiut 71515, Egypt; ^4^Department of Biostatistics and Epidemiology, Case Western Reserve University School of Medicine, Cleveland, OH 44106, USA

## Abstract

*Objective*. To study the reproductive outcomes of modified laparoscopic fimbrioplasty (MLF), a surgical technique designed to increase the working surface area of the fimbriated end of the fallopian tube. We postulated that an improvement in fimbrial function through MLF will improve reproductive outcomes. *Design*. Retrospective cohort study. *Setting*. Academic tertiary-care medical center. *Patients*. Women with minimal endometriosis or unexplained infertility, who underwent MLF during diagnostic laparoscopy (*n* = 50) or diagnostic laparoscopy alone (*n* = 87). *Intervention*. MLF involved gentle, circumferential dilatation of the fimbria and lysis of fimbrial adhesions bridging the fimbrial folds. *Main Outcome Measures*. The primary outcome was pregnancy rate and the secondary outcome was time to pregnancy. *Results*. The pregnancy rate for the MLF group was 40.0%, compared to 28.7% for the control group. The average time to pregnancy for the MLF group was 13 weeks, compared to 18 weeks for the control group. The pregnancy rate in the MLF group was significantly higher for patients ≤35 ys (51.5% versus 28.8%), but not for those >35 ys (17.6% versus 28.6%). *Conclusion*. MLF was associated with a significant increase in pregnancy rate for patients ≤35 ys.

## 1. Introduction

Ovum pickup occurs at midcycle when the dominant follicle, ovary, and fimbrial end of the tube interact behind the uterus near the cul de sac [1]. The factors that contribute to ovum pickup, besides tubal and fimbrial structure, have not been well characterized in humans but may include chemotactic factors, elastic mucoid projections from the fimbria, and peristaltic or pressure changes of the oviduct or surrounding ligaments [2, 3]. Our group designed a procedure designated as modified laparoscopic fimbrioplasty (MLF), to improve ovum pickup by the fimbria, to target and correct subclinical fimbrial lesions, and to increase the working fimbrial surface area.

For normal fallopian tubal function, the tube must be patent, in close proximity to the ovary, and freely mobile. Additionally, the fimbria must have sufficient surface area for ovum pickup. The fimbria needs to be closely apposed to the ovarian surface in order to capture ova from all sides; this can be visualized in the endoscopic video of ovulation [4].

Abnormal tubal function can be a factor in more than 20% of couples presenting with primary infertility [5]. An assessment of tubal architecture can be made with diagnostic laparoscopy, which can detect the presence of pelvic adhesions or endometriosis. However, laparoscopy is now less frequently a part of the standard initial evaluation for infertility. Falloposcopy, a technique that cannulates and visualizes the entirety of the fallopian tube via fiber optic imaging through the uterine cavity, is able to detect and target lesions, debris, and adhesions not visible or detectable with either HSG or diagnostic laparoscopy. Even though today this technique is less popular and is infrequently utilized, the findings from falloposcopy indicate that there is often more pathology present than is recognized by HSG or laparoscopy [3].

Currently, the empiric treatment for unexplained infertility often includes three cycles of ovarian stimulation with clomiphene citrate or gonadotropins with intrauterine insemination (IUI). If this approach is unsuccessful, couples can then proceed onto in vitro fertilization (IVF) or diagnostic laparoscopy [6].

The objective of this project was to examine the effectiveness of MLF, a novel, simple surgical technique that is performed during diagnostic laparoscopy in patients with minimal endometriosis or unexplained infertility. This study analyzed retrospectively the pregnancy rate and time to pregnancy after MLF. We hypothesized that fimbrial function is enhanced by MLF.

## 2. Materials and Methods

### 2.1. Study Design

This was a retrospective cohort study that compared the reproductive outcomes of women who underwent MLF during diagnostic laparoscopy to women who underwent diagnostic laparoscopy alone. The study was approved by the Institutional Review Board of University Hospitals Case Medical Center. The reproductive history, operative information, and reproductive outcomes of all subjects were collected from preexisting records. The primary outcome for this study was pregnancy rate, and the secondary outcome was time to pregnancy.

### 2.2. Subject Selection

Patients were identified from a database of 511 patients who had a laparoscopic procedure performed by the reproductive endocrine division between 2006 and 2012. The MLF group was comprised of women who underwent MLF during diagnostic laparoscopy performed by James H. Liu (JHL). The control group was comprised of women who underwent diagnostic laparoscopy without MLF by other members of our reproductive endocrine division. Other inclusion criteria were age less than 42 ys, diagnosis of either minimal endometriosis or unexplained infertility, and adequate follow-up while attempting to conceive.

Minimal, or Stage I, endometriosis was defined according to the American Society for Reproductive Medicine (ASRM) classification as superficial lesions less than 3 cm [7]. A diagnosis of unexplained infertility was given when a patient was infertile with normal ovulatory and tubal functions along with a normal sperm count for her partner. These were determined by the regularity of menstrual cycles, HSG, and semen analysis, respectively. When endometriosis lesions were minimal, they could be missed laparoscopically, resulting in patients being characterized as “unexplained infertility.” Thus, in many studies, minimal endometriosis and unexplained infertility were combined in the analysis.

Preoperatively, the two groups of patients were treated as patients of the entire reproductive endocrine division with similar preoperative treatment course. The cycles of Clomid and IUI were managed in the same fashion by the same group of nurses. The decision to proceed to laparoscopy was similar between the three group clinicians, typically after three unsuccessful cycles of Clomid and IUI. The only difference in their treatment was that JHL chose to perform the MLF and the other group clinicians did not.

Patients were excluded when other procedures were performed during the laparoscopy such as removal of an ectopic pregnancy, bilateral salpingectomy or salpingostomy, ovarian drilling, or myomectomy, as these might confound the effects of MLF on fertility by altering the woman's ability to conceive. Also, 10 subjects from each group were lost to follow-up. The final sample size was 50 MLF patients and 87 control patients. The study was powered for a minimum of 50 MLF patients and 57 control patients, to support a twofold increase in pregnancy rate from a baseline of 20% [8].

### 2.3. Surgical Procedure

The MLF procedure was performed during diagnostic laparoscopy. For the patients of JHL, MLF was performed as an additional procedure during the laparoscopy, regardless of the presence or absence of a visible pathology.

**Figure 1:**
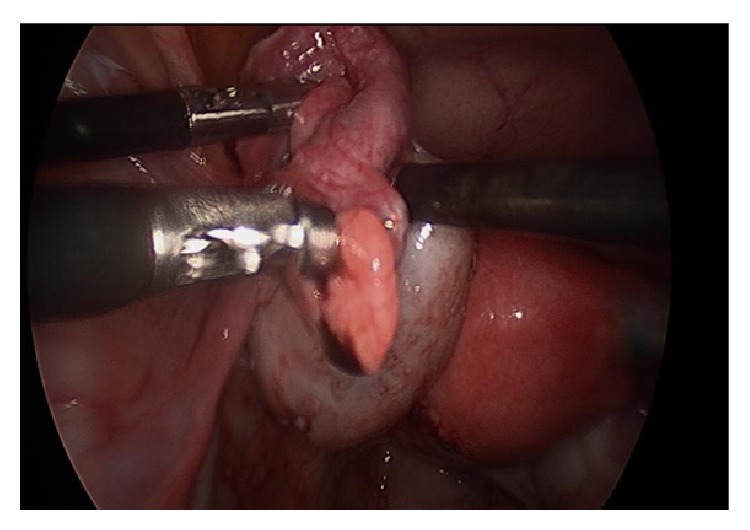
Endoscopic picture of MLF procedure; right angle cystic duct clamp inserted halfway into fimbria.

After a pelvic survey, two 5-mm accessory ports were placed, one in the right lower quadrant and the other in the left lower quadrant. If any evidence of endometriosis was noted, a small confirmatory biopsy was taken, and any minimal adhesions in the pelvis were lysed. The fimbriated end of each tube was then elevated with a nongrasping instrument, and dye was injected in order to more readily identify the tubal lumen. At the point of dye extrusion, a small right angle cystic duct clamp was inserted into the fimbrial lumen and gently opened 5–7 times with simultaneous rotation of the clamp to achieve circumferential dilatation in an attempt to expand the working surface area of the fimbria (Figure 1). This was then repeated with the opposite tube.

Before the procedure, intraluminal adhesions beyond the fimbriated end were not visualized; however, in some cases, intraluminal adhesions were noted once the clamp began dilating the fimbriated end. The adhesions that were noted at this time were not grossly blocking the tubal lumen but rather often bridged between the fimbrial folds. These intraluminal adhesions were not consistently documented and did not result in a change in the surgical or postoperative management.

### 2.4. Statistical Methods

Each patient's chart was reviewed for up to 70 weeks after her procedure or until she became pregnant. Each patient attempted to conceive after her procedure either naturally or with supplemental ovarian stimulation with various treatment protocols. These included clomiphene, gonadotropins, or combination clomiphene-gonadotropins, with or without intrauterine insemination. The choice of postsurgical treatment was a joint decision made by the patient and her physician. For women attempting to conceive with ovulatory stimulation, each cycle was tracked and recorded in a paper chart, including length and dose of treatment and ultrasound monitoring of endometrial thickness and number of codominant follicles, with a dominant follicle defined by a diameter of at least 15 mm averaged from all dimensions. Pregnancies were tracked through both paper and electronic charts by serum *β*hCG and ultrasound, with a positive serum *β*hCG result defined as greater than 10 IU/L. Eight MLF and 13 control patients chose to proceed to IVF after attempting to conceive with the above methods for an average of six months. The data from treatment cycles were included until IVF began.

The demographics of the two groups were analyzed by summary statistics and by univariate analysis. Survival analysis using Kaplan-Meier plots was used to display cumulative pregnancy rates for both groups and to determine the mean time to pregnancy. Number needed to treat (NNT) was calculated by the number of treatment cycles needed to achieve one pregnancy. For each outcome, a *p* value less than 0.05 (two-tailed) was used to determine significance. The subjects were further dichotomized into age groups, either ≤35 or >35; then the demographics and outcomes were compared for these age groups.

## 3. Results

### 3.1. Baseline Characteristics

The demographic features of the MLF and control cohorts are shown in Table 1. The two groups were similar in terms of age, BMI, smoking history, partner's smoking history, and length of relationship. There was a statistically significant difference in the ethnic distribution between the two groups, with a higher percentage of the MLF group reporting ethnicity as white (80.8% versus 60.2%, MLF versus control) and a lower percentage as black (6.4% versus 27.9%, *p* < 0.01). No differences in these characteristics were observed for women aged ≤35 ys when comparing the MLF group to the control group; however there was a significant difference in BMI, with a lower mean BMI in the younger MLF group when compared to the younger control group (24.9 versus 28.5, *p* < 0.01).

The baseline reproductive characteristics of the patients in both groups are also shown in Table 1. The two groups were comparable, with no significant differences in gravidity, day 3 FSH level, day 3 estradiol level, prior use of oral contraceptive pills, intraoperative chromopertubation findings, and semen analysis results.

For individual cycles that led to pregnancy and those that did not, the endometrial thickness (9.7 ± 2.6 mm versus 8.8 ± 2.6 mm, *p* = 0.07) and the number of dominant follicles (1.6 ± 1.2 versus 1.6 ± 1.0, *p* = 0.95) were comparable.

Univariate analysis examined the effect of each of the variables on the reproductive outcomes, namely, age, BMI, race, smoking history, FSH level, estradiol level, HSG findings, intraoperative chromopertubation findings, and semen analysis. None of these variables were found to have a significant impact on the outcome (data not shown).

Operative times were similar for the two groups (60.1 ± 17.7 minutes versus 62.6 ± 36.2 minutes, *p* = 0.59, MLF versus control). Minimal surgical complications were noted for each group (0% versus 3.5%, *p* = 0.29, MLF versus control).

### 3.2. Reproductive Outcomes

**Figure 2:**
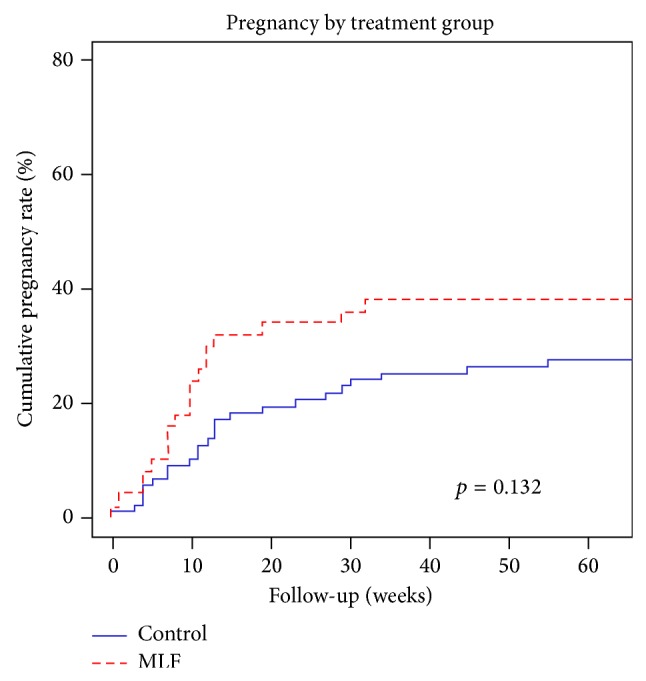
Pregnancy rate for MLF versus control over time (*p* = 0.13).

The Kaplan-Meier analysis of the cumulative pregnancy rate is shown in Figure 2. Overall, the pregnancy rate for the MLF group was 40.0%, compared to 28.7% for that of the control group (*p* = 0.13). The average time to pregnancy for the MLF group was 13.4 weeks compared to 18.4 weeks for the control group (*p* = 0.27) (Table 2).

**Figure 3:**
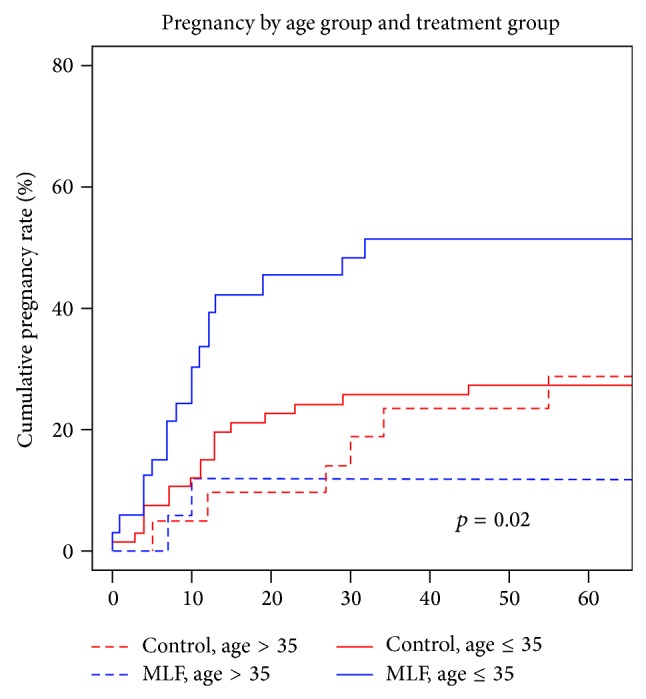
Pregnancy rate by treatment (MLF versus control) and age (women ≤35 ys, women >35 ys) over time. Significant difference for women ≤35 ys (*p* = 0.02), no significant difference for women >35 ys (*p* = 0.45).

Figure 3 shows the cumulative pregnancy rate with women dichotomized into two age groups with a cutoff of age 35 ys. This demonstrates that, for the younger cohort, a significantly higher percentage of the MLF group (*n* = 33) became pregnant compared to the control group (*n* = 66) (51.5% versus 28.8%, *p* = 0.02). However, there were no differences for women older than 35 years (17.6% versus 28.6%, *p* = 0.45). The average time to pregnancy for the MLF group in the younger cohort was 10.8 weeks compared to 15.7 weeks for the control group (*p* = 0.27).

The impact of ovulatory stimulation and IUI in postsurgical management was also evaluated. Couples in both groups often attempted to conceive multiple times with different methods. Analysis of treatment cycles indicates that the two groups, MLF versus control, attempted to conceive in comparable proportions without additional medication (16% versus 27%, *p* = 0.12), with clomiphene (48% versus 59.8, *p* = 0.18), and with gonadotropins (32% versus 26.4%, *p* = 0.49); however, a greater proportion of women in the MLF group attempted to conceive using combination clomiphene-gonadotropin therapy (36% versus 8.1%, *p* < 0.01).

For all conception cycles, MLF patients became pregnant without ovulatory stimulation 35% of the time compared to 28% of the time for the control group (*p* = 0.36). Both groups achieved pregnancy with timed intercourse 40% of the time and with IUI 60% of the time (*p* = 0.40).

For subjects aged ≤35 ys, the overall per cycle pregnancy rate was 22.7% for MLF compared to 13.3% for control (*p* = 0.06).

The NNT was calculated as the number of postsurgical cycles needed to achieve one pregnancy. The NNT for all women was 6 for the MLF group compared to 8 for the control group. For women aged ≤35 ys, the NNT for the MLF group was 4 compared to 8 for the control group.

## 4. Discussion

The present cohort study showed that MLF performed during routine diagnostic laparoscopy led to a significantly greater pregnancy rate of 51.5% for women ≤35 ys and a trend towards a higher pregnancy rate of 40.0% in the overall MLF cohort when compared to diagnostic laparoscopy alone. The MLF also showed a trend towards a shorter time to pregnancy: an average of 10 weeks for women ≤35 ys and 13 weeks for all women.

The per cycle pregnancy rate for MLF was 22% for women ≤35 ys and 16% for all women; this can be compared to the per cycle pregnancy rate of 32.4% with IVF for women of all ages with tubal infertility [9]. The NNT for the MLF group was 4 for women ≤35 ys and 6 for all women, which can be compared to the NNT of 3 for IVF for women of all ages and for women ≤35 ys with infertility of all causes, based on historical data [10].

The control and MLF groups were comparable for most demographic features, except for ethnic distribution for subjects of all ages and a lower BMI for the younger cohort. These observations can be expected given that the groups were not randomized. However univariate analysis determined that neither of these factors significantly impacted the outcome.

With regards to current treatment trends, the surgical role in the treatment of unexplained infertility has been largely supplanted by IVF. However, a variety of surgical options for optimizing tubal function are available, including neosalpingostomy, traditional fimbrioplasty, and our MLF. These surgical options can be an alternative or predecessor to IVF and are potential options for younger women.

A recent committee opinion article from the ASRM supports the role of tubal surgery as an alternative to IVF. The committee describes the benefits of tubal surgery for infertility, including that it is a one-time procedure, often minimally invasive, that one procedure allows multiple attempts at conception without additional treatment, and that it can result in multiple conceptions over time. The committee recommended proximal tubal cannulation and laparoscopic fimbrioplasty or neosalpingostomy to treat mild hydrosalpinges in young women without other pathology [9].

The MLF procedure differs significantly from the traditional procedure that is termed “fimbrioplasty.” The later, often associated with neosalpingostomy, is a similar, but distinct, technique, where visible periadnexal lesions are lysed, the tube is dilated with forceps, and fimbrial bridges are freed [11]. This procedure is indicated for patients with hydrosalpinx or known distal tubal obstruction secondary to pelvic infection or surgery. Modified fimbrioplasty (MLF) is used on patent tubes in patients with minimal endometriosis or unexplained infertility. Traditional fimbrioplasty targets visible lesions, while the MLF aims to correct subclinical lesions.

However one limitation of the current study is the nonrandomization of the initial surgery and the postsurgical treatments. Another limitation is the moderate loss to follow-up after the procedure as well as the modest size for the cohort groups. The conclusions would be stronger with a larger cohort size. Meanwhile, the comparability of the MLF and control groups, the comparability of the postsurgical treatment regimens, and the long follow-up period strengthen the study.

Given our promising preliminary results, the authors propose that the present described approach, MLF, would be an adjunctive procedure for women already undergoing diagnostic laparoscopy. It would be performed as an additional step during a routine diagnostic laparoscopy, similar to fulguration or excision of endometriosis or chromopertubation. At this point, the authors do not recommend undertaking diagnostic laparoscopy solely for the purpose of performing an MLF but rather only if there is already a plan to perform a laparoscopy.

## 5. Conclusion

MLF is a minimally invasive surgical technique that can be safely incorporated into diagnostic laparoscopy and may be an effective alternative to IVF for women with minimal endometriosis or unexplained infertility. The present findings need further validation by a larger, prospective, and randomized study to assess whether this procedure is still effective in a larger population and to assess whether the benefit extends to women over the age of 35 ys.

## Figures and Tables

**Table 1:** Demographic features of all subjects.

	All subjects			Age ≤ 35		
	MLF (*n* = 50)	Control (*n* = 87)	*p* value	MLF (*n* = 33)	Control (*n* = 66)	*p* value
Age	33.3 ± 6.5	32.2 ± 5.1	0.28	31.0 ± 2.9	30.1 ± 3.8	0.23
BMI	25.8 ± 5.5	28.2 ± 7.8	**0.04**	24.9 ± 4.6	28.5 ± 7.3	**<0.01**
Ethnicity						
White	38 (80.8%)	49 (62.0%)	**<0.01**	23 (76.7%)	37 (61.7%)	0.09
Black	3 (6.4%)	22 (27.9%)		3 (10.0%)	18 (30.0%)	
Others	6 (12.8%)	8 (10.1%)		4 (13.3%)	5 (8.3%)	
Smoking history						
Ever smoker	13 (26.0%)	26 (29.9%)	0.63	11 (33.3%)	21 (31.8%)	0.88
Never smoker	37 (74.0%)	61 (70.1%)		22 (66.7%)	45 (68.2%)	
Partner's smoking						
Ever smoker	15 (31.9%)	23 (27.7%)	0.61	10 (32.3%)	20 (31.8%)	0.96
Never smoker	32 (68.1%)	60 (72.3%)		21 (67.7%)	43 (68.2%)	
Length of relationship (years)	7.9 ± 5.1	6.8 ± 4.1	0.20	7.5 ± 5.8	6.3 ± 5.4	0.18
Gravidity						
0	24 (48.0%)	46 (52.9%)	0.59	36 (54.5%)	16 (48.5%)	0.57
≥1	26 (52.0%)	41 (47.1%)		30 (45.5%)	17 (51.6%)	
Day 3 FSH	57 ± 3.3	6.3 ± 3.2	0.39	6.0 ± 3.9	5.7 ± 2.3	0.78
Day 3 estradiol	45.1 ± 38.4	36.5 ± 27.1	0.22	50.7 ± 45.2	36.7 ± 26.6	0.11
Prior OCP use						
Yes	30 (78.9%)	64 (83.1%)	0.59	19 (73.1%)	47 (82.5%)	0.33
No	8 (21.1%)	13 (16.9%)		7 (26.9%)	10 (17.5%)	
Chromopertubation						
Patent	47 (97.9%)	80 (96.4%)	1.00	31 (96.9%)	61 (98.4%)	1.00
≥1 blocked	1 (2.1%)	3 (3.6%)		1 (3.1%)	1 (1.6%)	
Semen analysis						
Normal	15 (31.3%)	18 (26.1%)	0.54	12 (37.5%)	11 (22.0%)	0.13
Abnormal	33 (68.7%)	51 (73.9%)		20 (62.5%)	39 (78.0%)	

Note: MLF = women treated with modified laparoscopic fimbrioplasty. OCP = oral contraceptive pills. Data are expressed as mean ± SD for age, BMI, length of relationship, day 3 FSH level, and day 3 estradiol level. Data are expressed as numerator and percentage for all other factors (ethnicity, smoking history, gravidity, prior OCP use, chromopertubation findings, and semen analysis). *p * values were obtained by either Fisher's exact test or two-tailed *t*-test.

**Table 2:** Reproductive outcomes.

	All subjects			Subjects ≤ 35		
	MLF (*n* = 50)	Control (*n* = 87)	*p* value	MLF (*n* = 33)	Control (*n* = 66)	*p* value
Percent pregnant	20 (40%)	25 (28.7%)	0.13	17 (51.5%)	19 (28.8%)	**0.02**
Average time to pregnancy (weeks)	13.4	18.4	0.27	10.8	15.7	0.27

Note: MLF = women treated with modified laparoscopic fimbrioplasty. Data are expressed as numerator and percentage for the number and percent of women who conceived. Data are expressed as mean time to pregnancy for those women who became pregnant. *p* values were obtained by either Fisher's exact test or two-tailed *t*-test.

## Capsule

Improved pregnancy rate was observed in women ≤35 ys after modified laparoscopic fimbrioplasty.

## References

[1] Blandau R. J. (1978). Comparative aspects of tubal anatomy and physiology as they relate to reconstructive procedures. *Journal of Reproductive Medicine*.

[2] Eddy C. A., Pauerstein C. J. (1980). Anatomy and physiology of the fallopian tube. *Clinical Obstetrics and Gynecology*.

[3] Kerin J. F., Williams D. B., San Roman G. A., Pearlstone A. C., Grundfest W. S., Surrey E. S. (1992). Falloposcopic classification and treatment of fallopian tube lumen disease. *Fertility and Sterility*.

[4] Gordts S. (2008). *Human Ovulation Captured on Film*.

[5] The Practice Committee of the American Society for Reproductive Medicine (2012). Diagnostic evaluation of the infertile female: a committee opinion. *Fertility and Sterility*.

[6] Hatasaka H. (2011). New perspectives for unexplained infertility. *Clinical Obstetrics and Gynecology*.

[7] American Society for Reproductive Medicine (1997). Revised American Society for reproductive medicine classification of endometriosis: 1996. *Fertility and Sterility*.

[8] The Practice Committee of the American Society for Reproductive Medicine (2012). Endometriosis and infertility: a committee opinion. *Fertility and Sterility*.

[9] The Practice Committee of the American Society for Reproductive Medicine (2012). Committee opinion: role of tubal surgery in the era of assisted reproductive technology. *Fertility and Sterility*.

[10] Society for Assisted Reproductive Technology (2013). *Clinical Summary Report*.

[11] Yusoff Dawood M. (1999). Laparoscopic surgery of the fallopian tubes ana ovaries. *Seminars in Laparoscopic Surgery*.

